# Author Correction: High-contrast, synchronous volumetric imaging with selective volume illumination microscopy

**DOI:** 10.1038/s42003-022-03327-7

**Published:** 2022-04-11

**Authors:** Thai V. Truong, Daniel B. Holland, Sara Madaan, Andrey Andreev, Kevin Keomanee-Dizon, Josh V. Troll, Daniel E. S. Koo, Margaret J. McFall-Ngai, Scott E. Fraser

**Affiliations:** 1 Translational Imaging Center, University of Southern California, Los Angeles, CA 90089 USA; 2 Molecular and Computational Biology Section, University of Southern California, Los Angeles, CA 90089 USA; 3 Department of Biomedical Engineering, University of Southern California, Los Angeles, CA 90089 USA; 4 Pacific Biosciences Research Center, University of Hawaii at Manoa, Honolulu, HI 96822 USA

**Keywords:** Light-sheet microscopy, Wide-field fluorescence microscopy

Correction to: *Communications Biology* 10.1038/s42003-020-0787-6, published online 14 February 2020.

In this article the affiliation details for Kevin Keomanee-Dizon were incorrectly given as ‘Department of Biomedical Engineering, University of Southern California, Los Angeles, CA 90089, USA’ and ‘Translational Imaging Center, University of Southern California, Los Angeles, CA 90089, USA’ but should have been ‘Translational Imaging Center, University of Southern California, Los Angeles, CA 90089, USA’.

