# Publisher Correction: Author Correction: High-contrast, synchronous volumetric imaging with selective volume illumination microscopy

**DOI:** 10.1038/s42003-022-03474-x

**Published:** 2022-05-27

**Authors:** Thai V. Truong, Daniel B. Holland, Sara Madaan, Andrey Andreev, Kevin Keomanee-Dizon, Josh V. Troll, Daniel E. S. Koo, Margaret J. McFall-Ngai, Scott E. Fraser

**Affiliations:** 1 Translational Imaging Center, University of Southern California, Los Angeles, CA 90089 USA; 2 Molecular and Computational Biology Section, University of Southern California, Los Angeles, CA 90089 USA; 3 Department of Biomedical Engineering, University of Southern California, Los Angeles, CA 90089 USA; 4 Pacific Biosciences Research Center, University of Hawaii at Manoa, Honolulu, HI 96822 USA

**Keywords:** Light-sheet microscopy, Wide-field fluorescence microscopy

Correction to: *Communications Biology* 10.1038/s42003-022-03327-7, published online 11 April 2022.

This Correction added Andrey Andreev and Kevin Keomanee-Dizon as equally contributing authors together with Thai V. Truong, Daniel B. Holland, Sara Madaan, which was incorrect. This has now been reverted back to the original list of contributing authors.

