# Supplementary material for: High-contrast, synchronous volumetric imaging with selective volume illumination microscopy
Source: Commun Biol. 2020 Feb 14;3:74. doi: 10.1038/s42003-020-0787-6 (PMC7021898; doi:10.1038/s42003-020-0787-6)
Supplement: Supplementary file 8 — Supplementary Information [file 42003_2020_787_MOESM8_ESM.pdf]

Supplementary Information

Supplementary Figures

Supplementary Fig. 1	Schematic of the SVIM/SPIM optical setup, and description of operation procedure
Supplementary Fig. 2	Imaging of sub-diffractive fluorescent beads to characterize resolution of SVIM
Supplementary Fig. 3	SVIM offers improved contrast over conventional wide-field LFM
Supplementary Fig. 4	Simulation of SVIM contrast degradation with increased background noise
Supplementary Fig. 5	Enhanced effective resolution enabled by the higher contrast of SVIM
Supplementary Fig. 6	SVIM enabled single-bacterium-resolution imaging and quantification of 3D bacterial flow tracks around the squid light organ
Supplementary Fig. 7	Contrast enhancement of SVIM in imaging the zebrafish beating heart and blood flow
Supplementary Fig. 8	Zoomed-in, 3D-rendered views of the zebrafish spontaneous brain activity
Supplementary Fig. 9	Light-evoked brain response captured by 2p-SVIM

Supplementary Tables

Supplementary Table 1	Part numbers and descriptions of key components of optical setup
Supplementary Table 2	Imaging and reconstruction parameters for all presented results
Supplementary Table 3	Volumetric imaging rate normalized by the number of diffraction-limited voxels

Supplementary Notes

Supplementary Note 1	Discussion on implementations of selective volume illumination
Supplementary Note 2	Volumetric imaging rate normalized by the number of diffraction-limited voxels

Supplementary Figures

Supplementary Figure 1

Schematic of the SVIM/SPIM optical setup, and description of operation procedure

Top-view of the optical setup, depicting only the key components. (See Supplementary Table 1 for specific part numbers of the key components.) Laser beams, for 1-photon (1p) and 2-photon (2p) excitation, were directed by silver mirrors (MR) and combined into the same optical path by a polarizing beamsplitter (BS). The laser light was routed into a 2-dimensional scanning galvos module (GV), then through the scan lens (SL) and illumination tube lens (TL-I), and into the illumination objective (IO). Sample (S) was suspended from the top of the liquid-filled sample chamber (SC), and the detection objective (DO) collected the fluorescence light generated at the sample. The y direction of the experimental coordinate system is anti-parallel to gravity. The beam at GV was imaged to the back focal plane of IO by SL and TL-I, so adjusting the rotational positions of the galvos enabled translating the illumination beam at the sample in the y and z directions.

Fluorescence signal collected by the DO passed through the appropriate interference bandpass filters (FT) to block the laser light and select for the right detection colors. The fluorescence was recorded by a detection module that allowed imaging in either SVIM or SPIM mode. In SVIM mode, as depicted in the figure, detection tube lens (TL-D) formed the image of the sample at its focal plane, where the micro-lens array (LA) was placed. The desired light-field image was formed at focal plane F, at a distance f away from the LA (where f = focal length of the micro-lens). A pair of identical photographic lenses (RL-1 and RL-2), configured in 4f mode, relayed the light-field image at F to be recorded at detection camera (DC). The optical parameters of the LA and the DO and TL-D, for the different imaging magnifications of 32X and 20X (see Supplementary Table 2), were chosen to ensure that the spatial-angular bandwidth of the light-field collected by the DO was matched to that of the LA[1,2]. To operate in SPIM mode, LA was moved entirely out of the optical path, and the entire block of (RL-1, RL-2, and DC) was moved by distance f closer to the TL-D (along the –z direction). Computer-controlled motorized translational stages (not shown) were used to allow convenient and reproducible switching between SVIM and SPIM modes.

Not shown was an imaging module looking at the sample from opposite to the IO, which allowed observation of the laser illuminated sample region in the yz plane. This imaging module was used to verify and calibrate the galvos scanning parameters to achieve the desired SVI. Also not shown was a separate beam path that directed the illumination laser light toward the sample along the +z direction – this path was used for wide-field illumination of the sample.

For 32X (& 20X) imaging with 1p excitation, the illumination beam size was adjusted to achieve an NA of ~ 0.04 (& 0.03), which following Gaussian-beam optics[9] yielded excitation depth of focus of ~ 250 μm (& 470 μm) and FWHM focused beam waist of ~ 5 μm (& 6 μm). For 20X imaging with 2p excitation, the NA was ~ 0.04, yielding excitation depth of focus of ~ 500 μm and FWHM focused beam waist of ~ 6 μm.

During SPIM imaging, the illumination beam was scanned in the y direction, resulting in a scanned light sheet transecting the sample in the xy plane, and the sample was moved along z to achieve 3D imaging. During SVIM imaging with 2p excitation, the beam was scanned in both the y and z directions, with appropriate amplitudes, to paint out the desired selectively-illuminated volume at the sample (galvo line scan rate = 1 kHz along y, and galvo stepping along z). During SVIM imaging with 1p excitation, a removable cylindrical beam-shaping module was put in place, up-stream of the GV, where cylindrical lenses (CL-1) and (CL-2) expanded the beam along y-direction by 2x, and cylindrical lens (CL-3) focused the beam along the y-direction at the GV. This focusing resulted in a static illumination light sheet (in the xy plane) at the sample, and the beam only needed to be scanned along one direction, z, to enable painting out the desired selectively-illuminated volume (galvo line scan rate = 1.5 kHz). A custom-written LabView program, in conjunction with the software Micro-Manager[3], coordinated the galvos scanning and camera triggering to ensure that within one camera exposure the volume of interest was illuminated an integer number of times, or that it was illuminated > 10 times, to ensure that the excitation intensity was uniform to better than 10% from frame to frame. Alternative selective volume illumination approaches are discussed in Supplementary Note 1.

Supplementary Figure 1

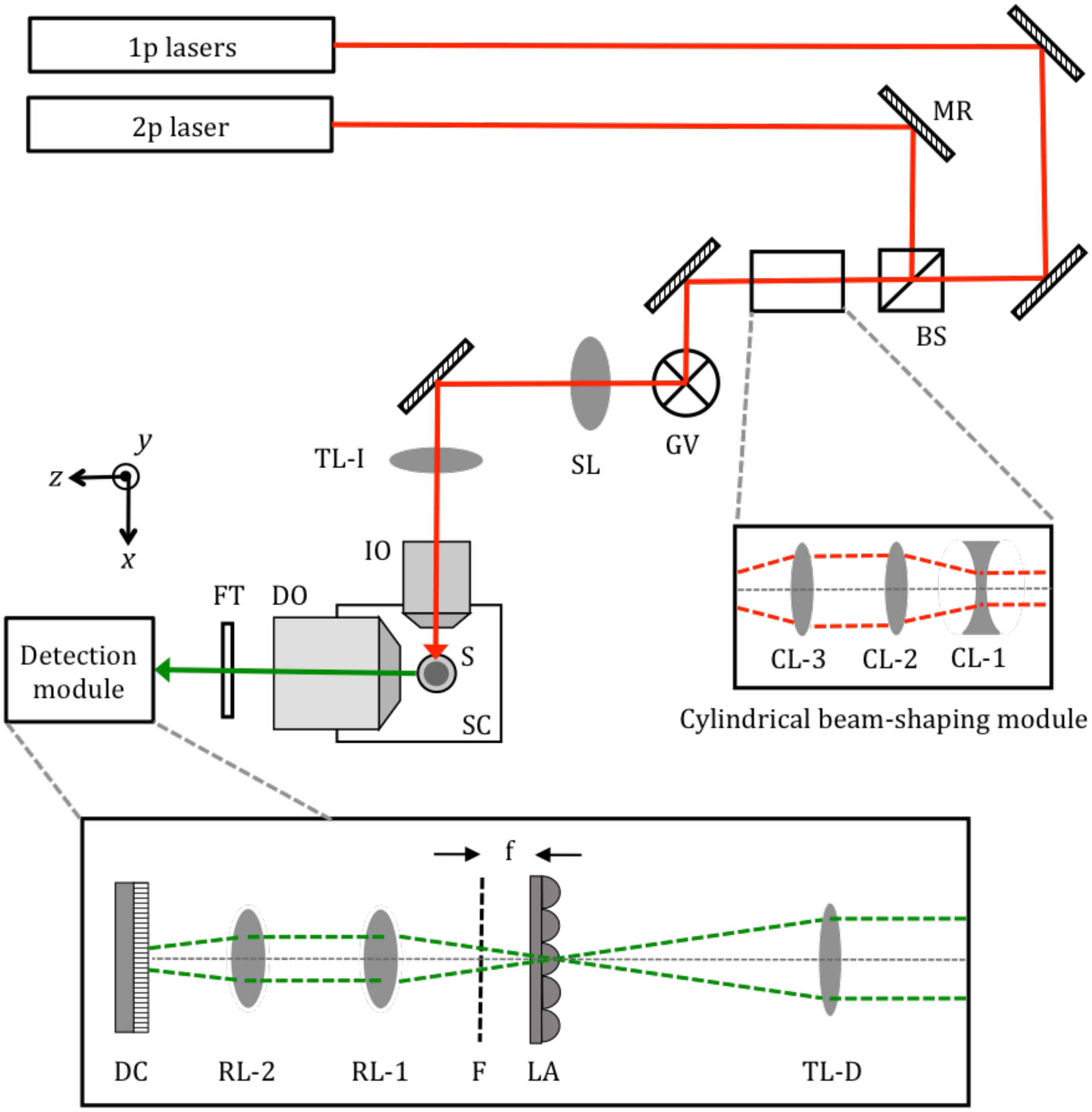

Supplementary Figure 2

Imaging of sub-diffractive fluorescent beads to characterize resolution of SVIM

Resolution performance of SVIM was characterized by imaging sub-diffractive fluorescent beads (diameter = 175 nm, PS-Speck, ThermoFisher) embedded in low-melting agarose. **(a)** 3D-rendered and **(b)** maximum projection view of the reconstructed volume of beads, spanning ~ 400 x 400 x 100 (x,y,z) μm^3. We sampled the point-spread-function (PSF) of multiple beads across the field of view as indicated by the yellow boxes shown in **(b)**. Line profiles along the lateral and axial directions **(c)** show a nominal Gaussian-fitted full-width-half-maximum (FWHM) resolution of 3 μm laterally and 6 μm axially **(d)**. This resolution performance was as expected for the optical parameters used (see Methods). Scale bars, 50 μm.

Supplementary Figure 2

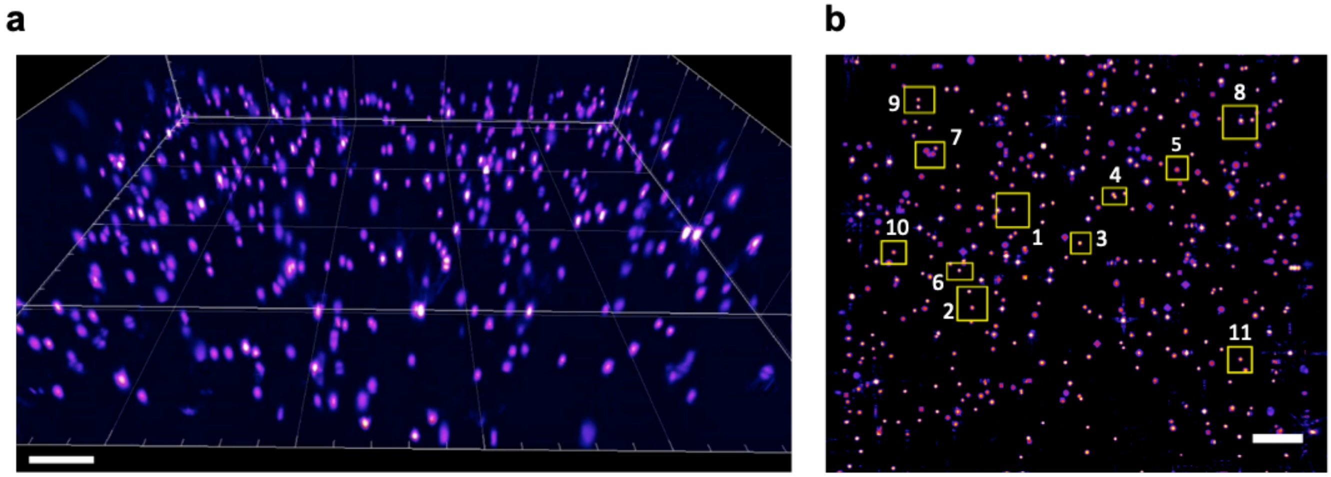

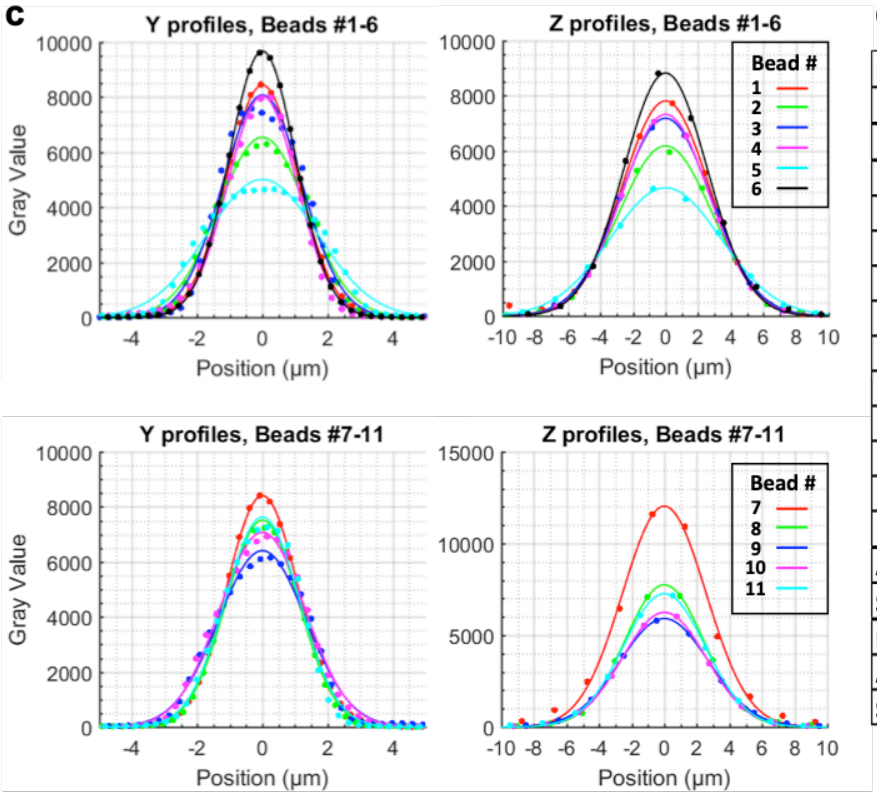

d

Bead Profile Fit Results			
FWHM (μm)			
Bead #	X	Y	Z
1	2.56	2.57	6.26
2	2.94	3.18	6.73
3	2.95	2.85	6.52
4	2.72	2.49	6.30
5	4.05	3.98	7.98
6	2.26	2.39	6.03
7	2.55	2.58	6.04
8	2.90	2.61	5.71
9	3.15	3.17	6.40
10	3.08	3.08	6.03
11	2.80	2.67	5.91
Avgerage	2.91	2.87	6.35
Std. Dev.	0.46	0.46	0.61
For all X, Y together,			
Average:	2.89		
Std. Dev.:	0.45		

Supplementary Figure 3

SVIM offers improved contrast over conventional wide-field LFM

Images of the same sub-volume of the GFP-labeled vasculature of a 5-dpf, *Tg(kdrl:eGFP),* zebrafish larva captured with **(a)** SPIM, and **(b-e)** SVIM with z-extents of 100, 200, 300, and 550-μm, respectively, and **(f)** wide-field LFM. A subset of images in **(a-e)** are shown in Fig. 2a. See Fig. 2b for quantitative analysis of the contrast obtained with SVIM of increasingly smaller z-extents having increasingly higher contrast, approaching the performance of SPIM, and far exceeding the image quality obtained with wide-field illumination. The images shown are each averaged-intensity z-projections of the same 40-μm thick sub-volume, centered at approximately 170 μm into the specimen, with the image intensity normalized to full scale with 0.4% saturation. In **(g, h)** we demonstrate that the lower background and higher contrast achieved with SVIM helped to produce reconstructed images that were more similar to the "ground-truth" SPIM images. The z-projected images shown in **(a-e)** were overlaid in the lateral plane, as shown in **(g)** for the 2 cases of SPIM and 100- μm SVIM, and intensity line profiles were compared between all the imaging modalities, along two representative lines that cross multiple blood vessels **(h)**. Overall, we found good semi-quantitative correspondence between SPIM and SVIM in capturing the vascular structures. Importantly, this correspondence was generally better for SVIM as compared to conventional wide-field LFM; the correspondence improved as the illumination extent was reduced. Full quantitative correspondence between the light-field reconstructed images and SPIM is not expected, due to SVIM's inherent lower and non-uniform resolution, and artifacts/limitations in the computational nature of LFM in general[1,2]. Active research efforts are on-going to fully characterize and improve the correspondence between LFM and image ground truth[4,5]. Scale bar, 100 μm.

Supplementary Figure 3

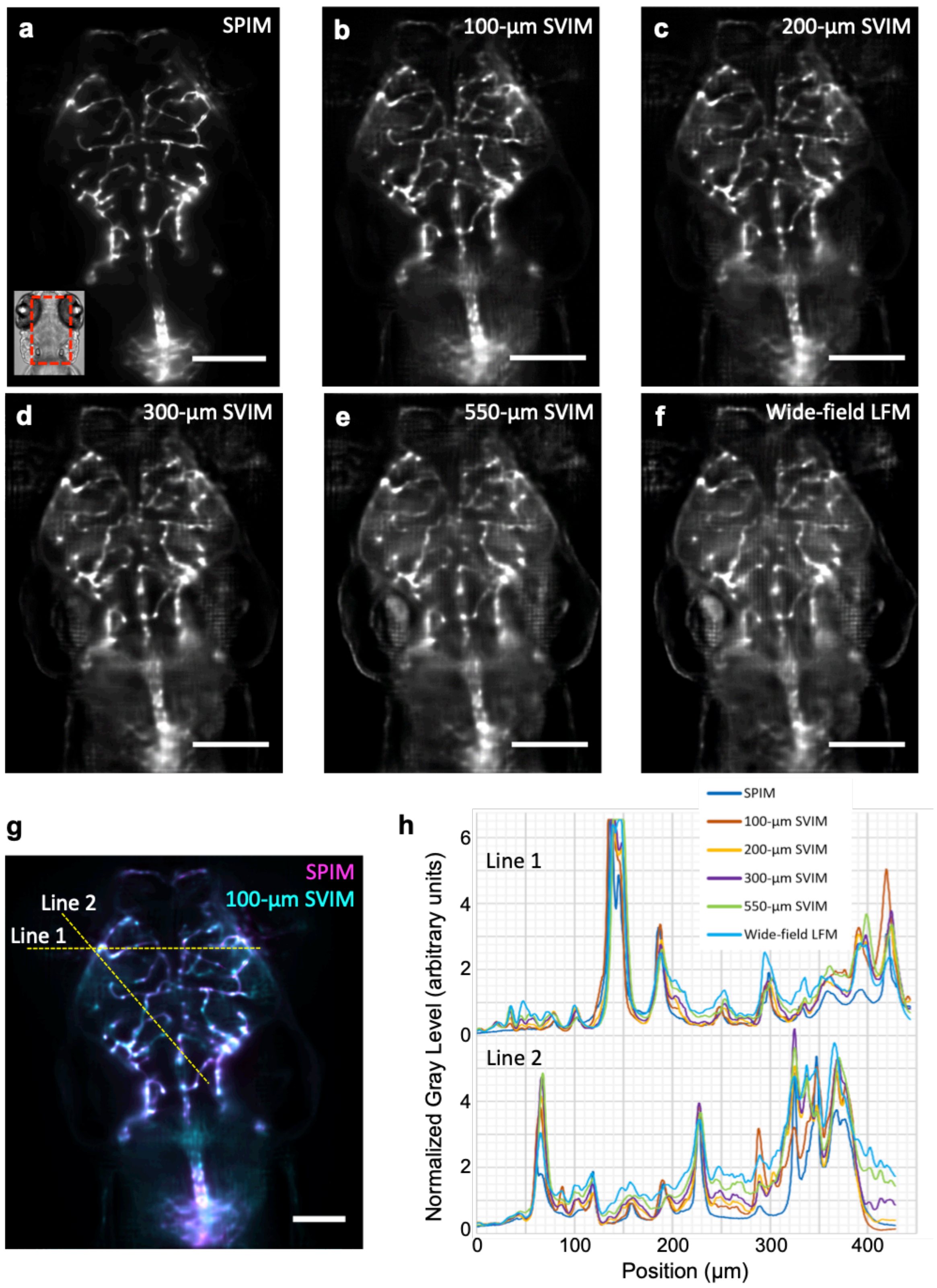

Supplementary Figure 4

Simulation of SVIM contrast degradation with increased background noise

Averaged-intensity z-projection images of the same 40 μm-thick sub-volume of GFP-labeled vasculature of a 5-dpf zebrafish larva, centered at approximately 170 μm into the specimen (as in Fig. 1d), comparing **(a)** SPIM, **(b)** 100-μm SVIM, **(c)** 300-μm SVIM, and **(d, e)** 100-μm SVIM with different levels of Poisson noise applied to the raw light-field images before reconstruction, to simulate the extra background signal that would be expected to come from larger illumination extents (λ = 8,400 corresponds to the mean photon number per pixel of the raw light-field image of 300-μm SVIM; λ = 10,000 and 13,000 simulates even larger illumination extents). See Methods for details of simulation. The images in **(a-e)** show that applying noise to 100-μm SVIM led to both increased background and artifacts in the reconstructed volumes, which was consistent with our empirical results (Fig. 2a, Supplementary Fig. 3). Comparison of the quantitative contrast of the 3D images is shown in **(f)**, similar to the comparison shown in Fig. 2b. For each modality, contrast was measured at every available z-plane and normalized against the top plane (z = -50 μm) of SPIM. As expected, 100-μm SVIM with increasing levels of noise applied (λ = 8,400, 10,000, and 13,000) had poorer contrast than 100-μm SVIM. Note that when $\lambda > 10{,}000$, 100-μm SVIM exhibited even lower contrast than 300-μm SVIM. Scale bars, 100 μm.

Supplementary Figure 4

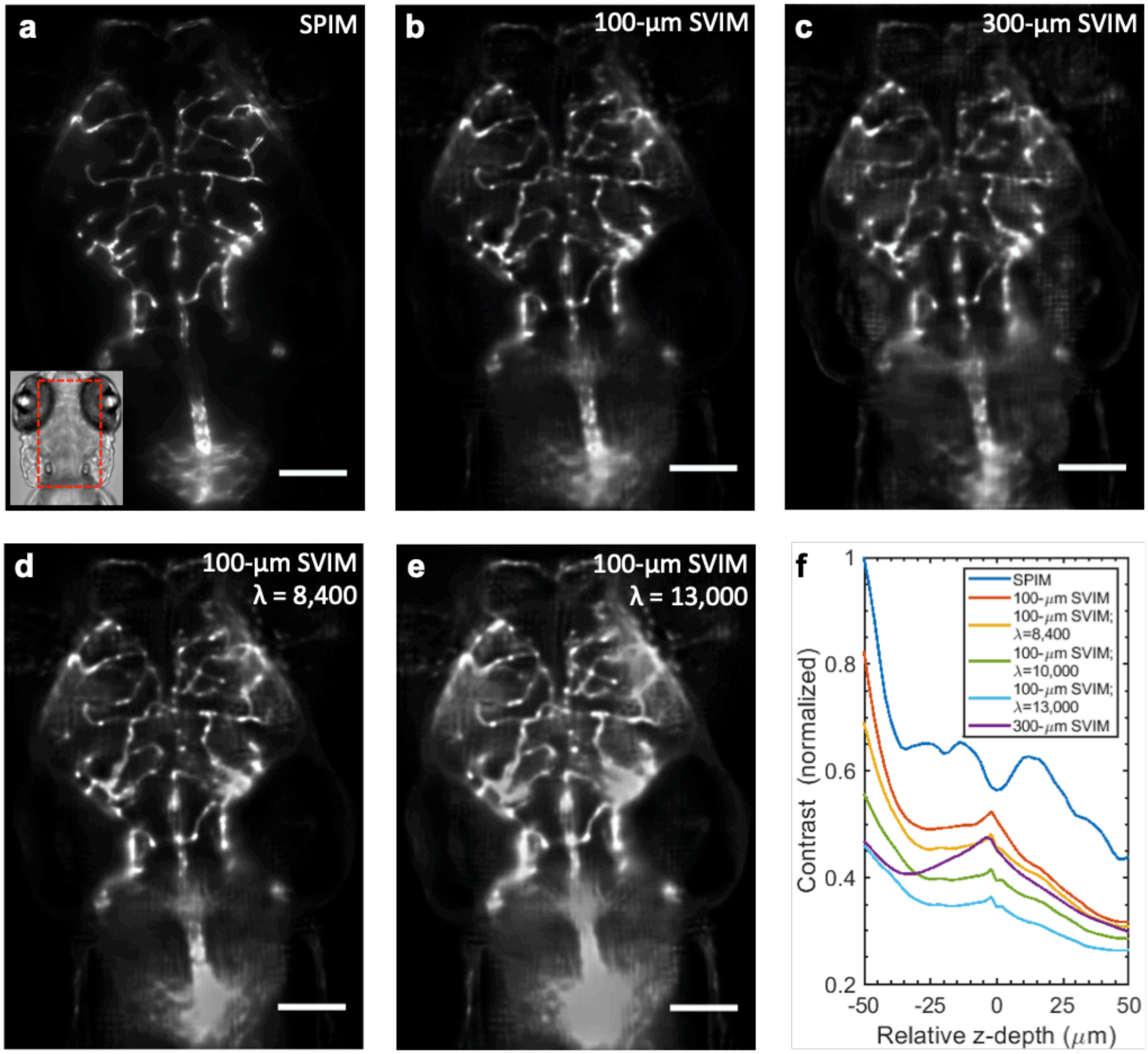

Supplementary Figure 5

Enhanced effective resolution enabled by the higher contrast of SVIM

To compare the effective resolution achieved with the various imaging modalities, we quantified the full-width-half-maximum (FWHM) diameter of the same blood vessels captured by each modality. Starting in the SPIM 3D dataset, we selected a single z-slice that had four well-imaged blood vessels of approximately the same size. We then found the matched z-slices in the SVIM and LFM datasets. Shown in **(a-f)** are the corresponding single z-slice images, selected at z-depth = 150 μm into the sample (50 μm above the native focal plane) across all modalities. Similar to all presented results, SPIM images were background-subtracted to account for camera dark-counts and SVIM images were background-adjusted within the reconstruction process. Four rectangular regions-of-interest (ROI), labeled 1-4, were generated by a MATLAB script to traverse orthogonally the respective blood vessels, whose zoomed-in images are shown in **(g)**. Each rectangular ROI had length of ~ 50 μm and width of ~10 μm, and the line profiles were calculated as averages across the width extent. All line profiles were normalized to peak value equal to 1, from which the mean FWHM and standard deviation values were measured and quantified in **(h)**. SPIM, as expected, exhibited the best resolution, establishing the ground-truth mean FWHM diameter of ~ 5 μm for the analyzed blood vessels. With SVIM, smaller selectively-illuminated volume extents yielded decreased background and increased effective resolution, represented by the achieved mean FWHM diameter of the blood vessels, showing an improvement of ~ 35% between 100-μm SVIM and wide-field LFM. Scale bars, **(a-f)** 100 μm, **(g)** 30 μm.

Supplementary Figure 5

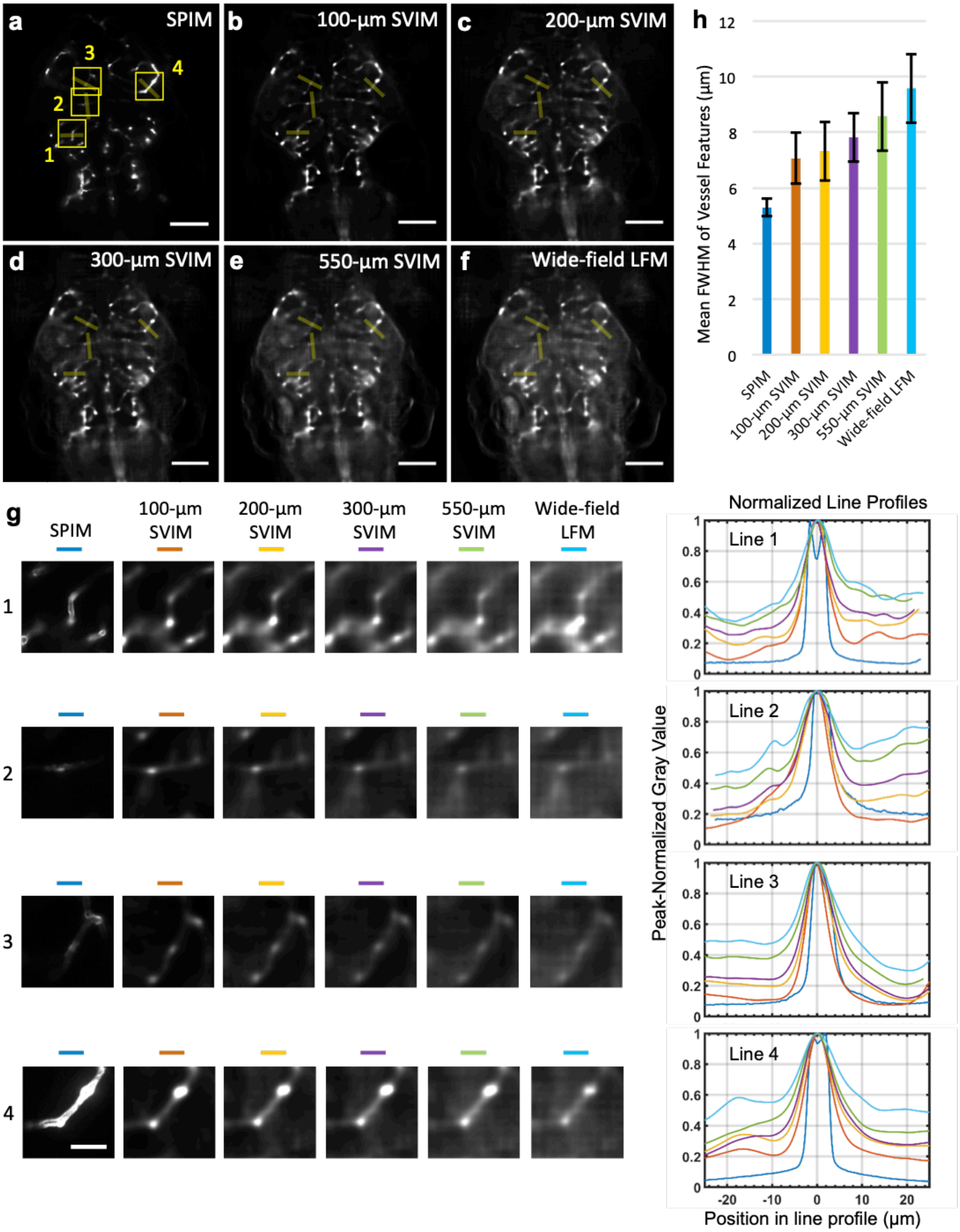

Supplementary Figure 6

SVIM enabled single-bacterium-resolution imaging and quantification of 3D bacterial flow tracks around the squid light organ

(a) Photograph of a representative juvenile Hawaiian bobtail squid, *Euprymna scolopes*, with inset showing the light organ area, which will be selectively colonized by the bacteria *Vibrio fischeri* as part of the symbiosis between the two organisms. **(b)** 3D-rendered, maximum-intensity projection view of a 200-μm z-stack taken with SPIM, over the approximately same region of interest as captured with light-field imaging. SPIM imaging speed was at 6 z-slices/s or 0.035 volumes/s. SVIM allowed synchronous volumetric imaging speed of 20 volumes/s to capture more accurately the 3D flow field across the z-depth **(c)** (duplicated here from **Fig. 2c**), and enabling quantification of the speed of individual bacterium flow tracks **(d)**. See Supplementary Table 2 for full imaging and reconstruction parameters. Scale bar, 100 μm.

Supplementary Figure 6

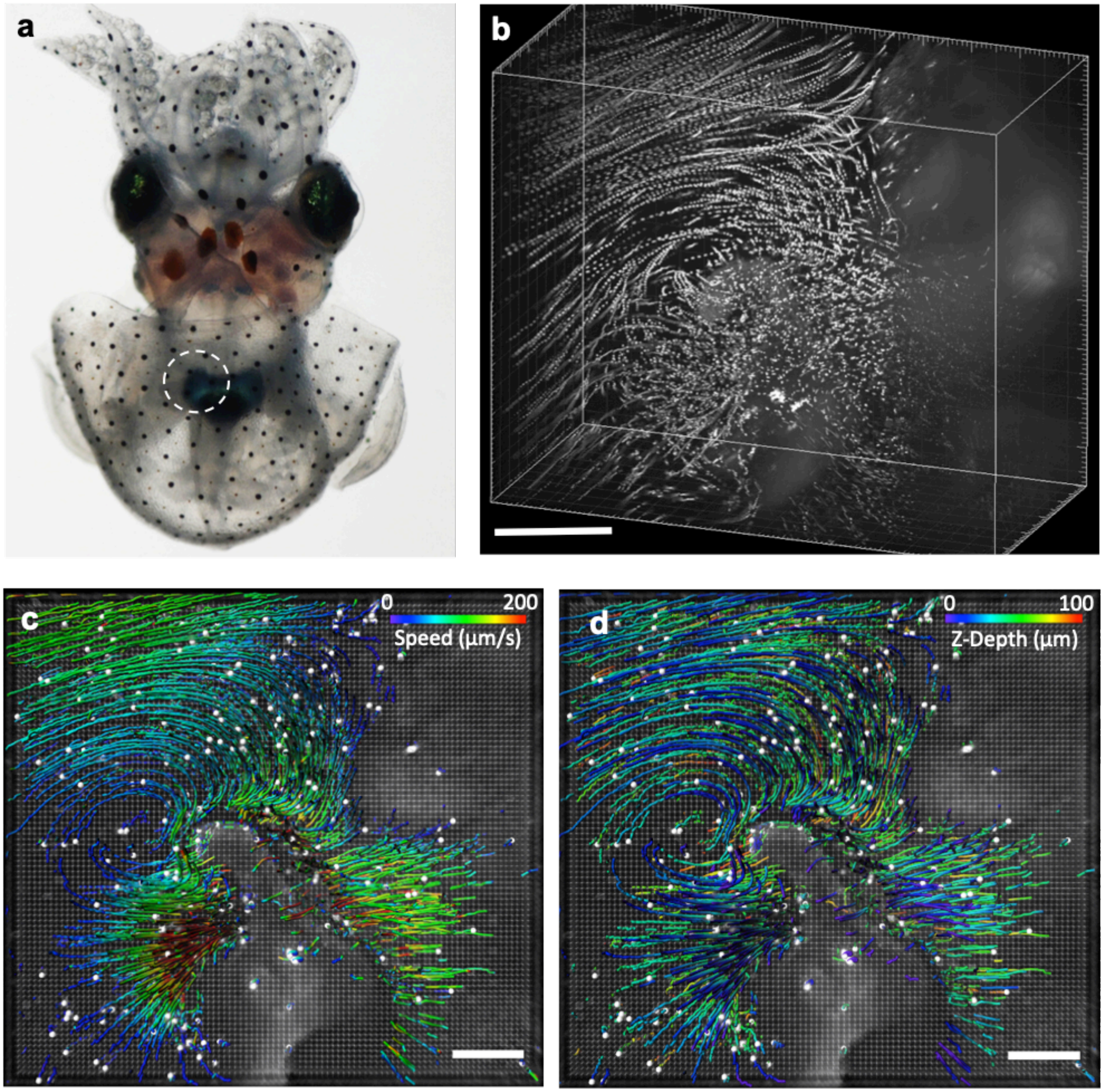

Supplementary Figure 7

Contrast enhancement of SVIM in imaging the zebrafish beating heart and blood flow

Comparison of image reconstructions acquired with SVIM and wide-field LFM, of fluorescently-labeled endocardium and blood cells in the beating heart of a 5-dpf zebrafish larva, captured at 90 volumes/s. See Supplementary Table 2 for details on imaging and reconstruction parameters. Taking advantage of the periodic nature of the beating heart, movies taken with SVIM and wide-field LFM were frame-synchronized to depict a similar sequence of the beating motion, to facilitate frame-to-frame comparison of image quality. Shown in **(a, b, d, e)** are the averaged-intensity z-projections of the 3D reconstructed z-stack at the time points as labeled, where t = 0 ms corresponded to when the atrium was maximally contracted. See Supplementary Movies 6 and 7 for the full temporal sequences that spanned several heart beats. Comparison of the quantitative contrast in imaging the endocardium and blood cells were shown in **(c)** and **(f)**, respectively. Image processing and contrast analysis were carried out with procedure similar to that used for the vasculature results, Fig. 2b (Methods). The contrast was calculated for the averaged-intensity z-projection images as shown in **(a, b, d, e)**, for each time point of the 50-frame beating cycle, and normalized to the averaged value of the wide-field LFM case. SVIM achieved approximately 50% and 10% higher contrast than wide-field LFM over the entire beating cycle, in imaging the endocardium and blood cells, respectively. Scale bar, 50 μm.

Supplementary Figure 7

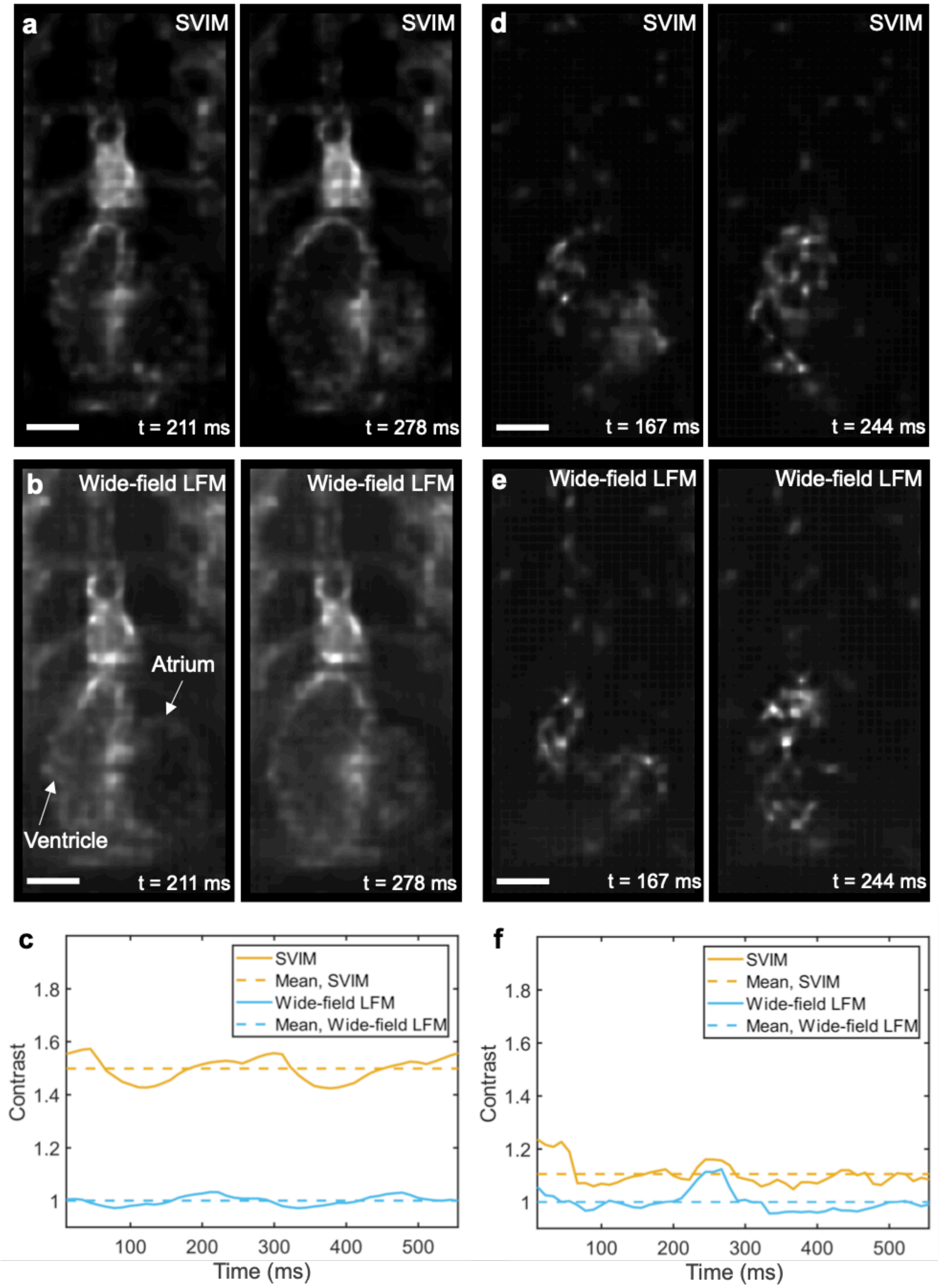

Supplementary Figure 8

Zoomed-in, 3D-rendered views of the zebrafish spontaneous brain activity

Higher magnification, 3D-rendered views from the functional imaging of a 5-dpf zebrafish's spontaneous brain activity (same results as in Fig. 4a-c). Shown in **(a)** is the 3D-rendered view of the time-domain standard-deviation-projection of spontaneous brain activity over a time window of 100 s, of the entire imaged volume, captured with 2p-SVIM. **(b-d)** Zoomed-in views of approximately the same region of the zebrafish brain for 2p-SVIM, 1p-SVIM, and wide-field LFM, respectively. Spot segmentation of the time-domain standard-deviation-projections was carried out to identify neurons that were active during the experimental time window (see Methods for full details on the processing and analysis of the neuroimaging results). The segmented active neurons are depicted as cyan-colored ellipsoids in the image panels. Image contrast has been manually adjusted for each image panel to improve visualization of individual neurons. Scale bars, **(a)** 100 μm, **(b-d)** 20 μm.

Supplementary Figure 8

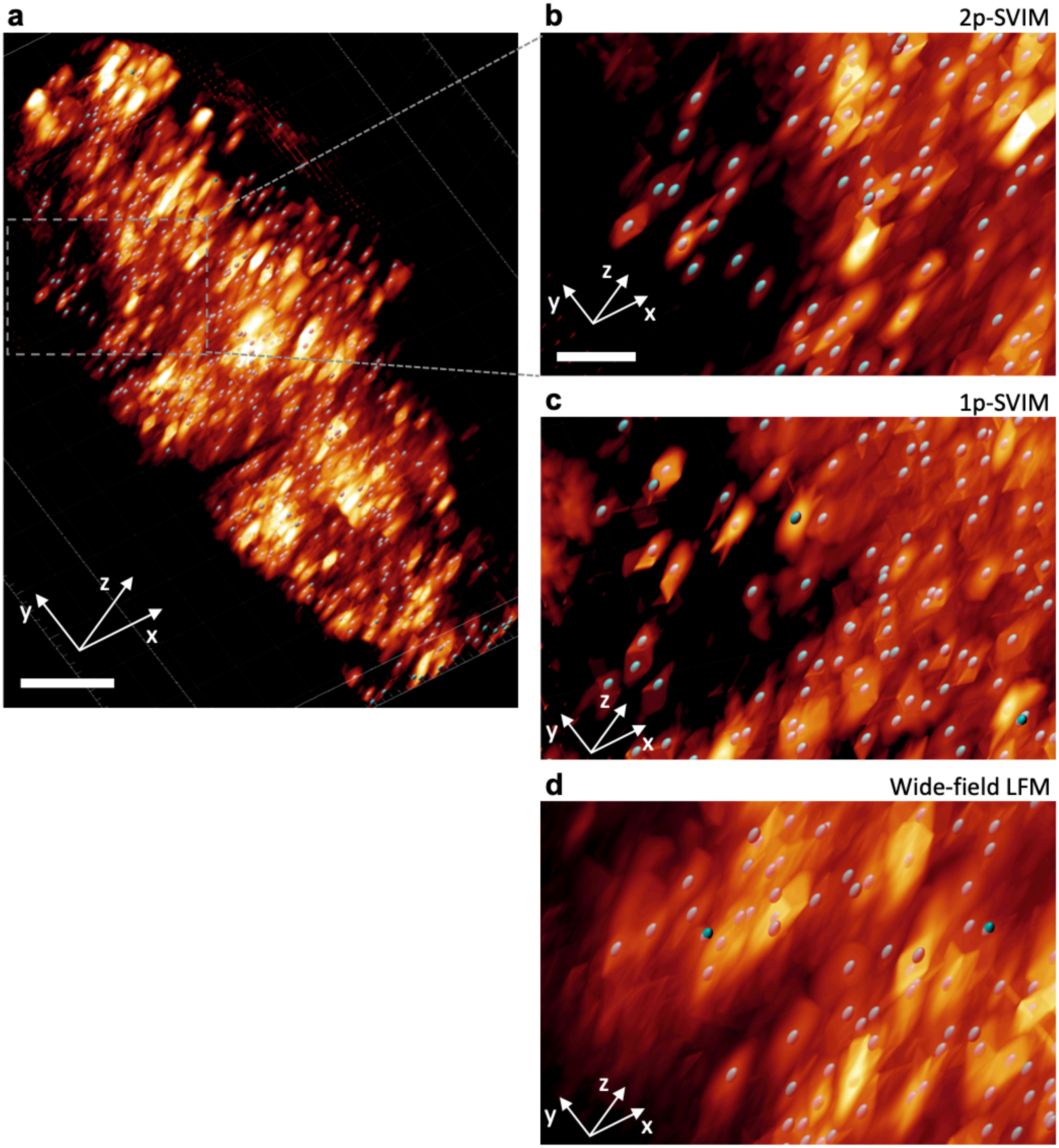

Supplementary Figure 9

Light-evoked brain response captured by 2p-SVIM

Functional imaging of a 5-dpf larval zebrafish with pan-neuronal fluorescent calcium indicators, *Tg(elavl3:H2b-GCaMP6s)*, across a time window of 100 s with a LED light turned on at the midpoint. Analysis and presentation of the imaged results are similar to the spontaneous activity window shown in Fig. 4. Cellular resolution representations of active neurons were found with standard methodology based on spot segmentation of the time-domain standard deviation of the 3D time series data (Methods). Images shown in **(a-c)** are depth color-coded of the time-domain standard-deviation-projection of the light-field reconstructed 3D time series data, where colored puncta represent active neurons. Activity traces of segmented neurons shown in **(d-f)** were further grouped by k-means clustering. Only the results recorded with 2p-SVIM revealed a group of neurons (marked by *), that exhibited a clear response to the LED light turning on at t = 50 s. Scale bar, 100 μm.

Supplementary Figure 9

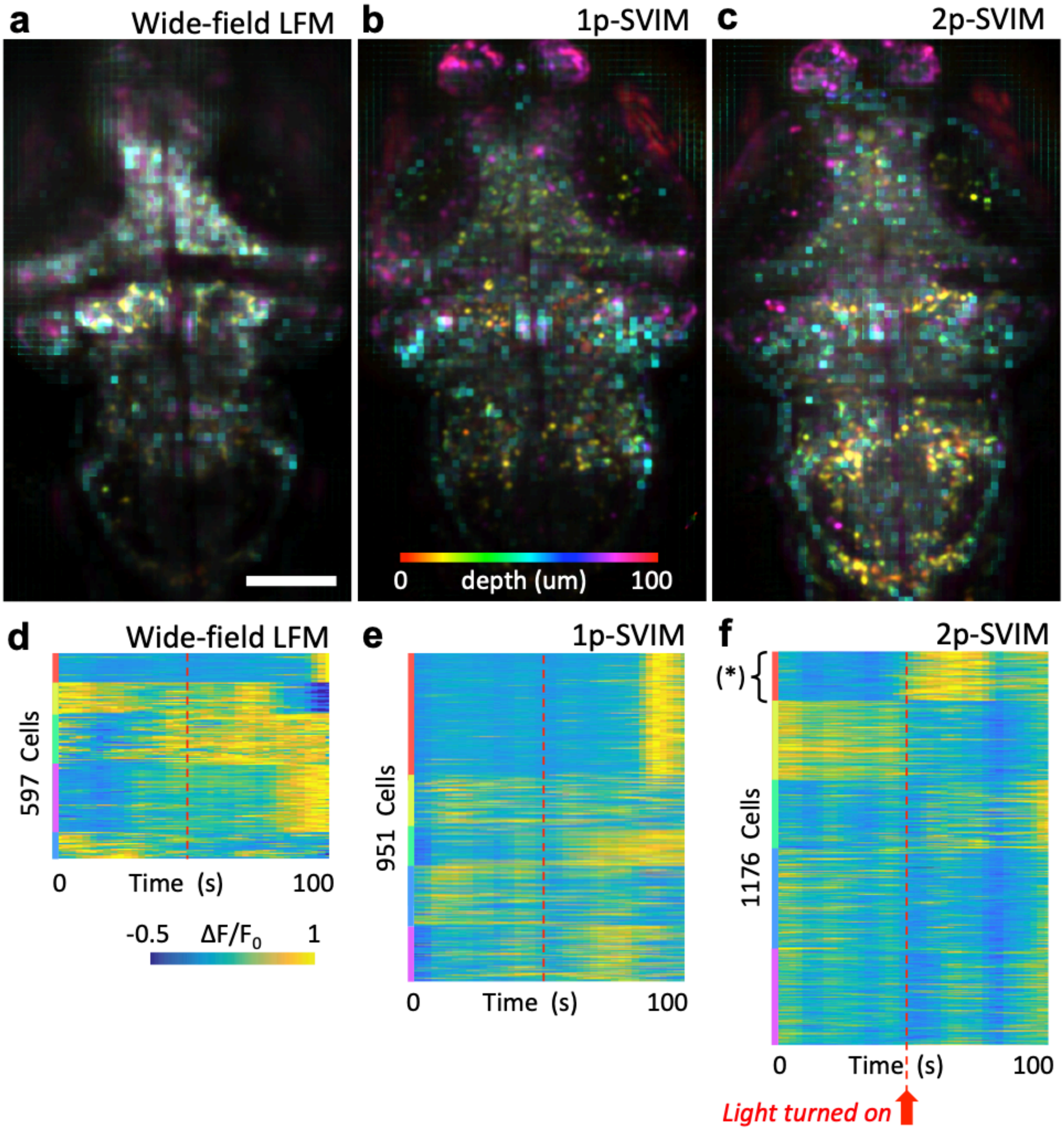

Supplementary Tables

Supplementary Table 1

Part numbers and descriptions of key components of optical setup

Item	Acronym (Sup. Fig. 1)	Detailed descriptions	Part number	Manufacturer/ Vendor
Laser system, visible 1-photon excitation	1p lasers	SOLE-6 Light Engine (lines used: 488, 561 nm)	SOLE-6 Light Engine	Omicron
Laser, near-infrared 2-photon excitation	2p laser	Chameleon Ultra 2	Chameleon Ultra 2	Coherent
Silver mirrors	MR		PF10-03-P01	Thorlabs
Polarizing beamsplitter	BS	Broadband, 620-1000 nm	PBS102	Thorlabs
Cylindrical lens	CL-1	f = -75 mm	LK1432RM-A	Thorlabs
Cylindrical lens	CL-2	f = 150 mm	LJ1629RM-A	Thorlabs
Cylindrical lens	CL-3	f = 100 mm	LF1567RM-A	Thorlabs
Galvos system, 2-dimensional	GV	6-mm aperture, silver mirrors	H8363	Cambridge Tech.
Scan lens	SL	f = 110 mm	LSM05-BB	Thorlabs
Tube lens, illumination	TL-I	f = 200 mm	AC508-200-B	Thorlabs
Illumination objective, 10X	IO	NA = 0.3, f = 20 mm, water immer., WD = 3 mm	CFI Plan Fluor 10X W	Nikon
Sample chamber	SC	Machined from custom-design; black delrin	N/A	Proto Labs
Detection objective 16X	DO	NA = 0.8, f = 12.5 mm, water immer., WD = 3 mm	CFI75 LWD 16X W	Nikon
Detection objective 20X	DO	NA = 0.5, f = 9 mm, water immer., WD = 3.5 mm	UMPLFLN 20XW	Olympus
Filter, detection (green fluorescence)	FT	Pass band = 525/50 nm; 25-mm diameter	FF03-525/50-25	Semrock
Filter, detection (red fluorescence)	FT	Pass band = 593/46 nm; 25-mm diameter	FF01-593/46-25	Semrock
Tube lens, detection (used with 16X DO)	TL-D	f = 400 mm	AC508-400-A	Thorlabs
Tube lens, detection (used with 20X DO)	TL-D	f = 180 mm	AC508-180-A	Thorlabs
Micro-lens array	LA	Pitch = 150 μm, f = 3.0 mm, area = 16 × 16 mm^2	APO-Q-P150-F3.0(633)	Flexible Optical B.V.
Relay lens	RL-1, RL-2	f = 50 mm; f/1.4	NIKKOR	Nikon
Detection camera	DC	Scientific CMOS, pixel size = 6.5 μm, 2560 × 2160 pixels	Zyla 5.5	Andor

f: focal length
NA: numerical aperture
WD: working distance

Supplementary Table 2

Imaging and reconstruction parameters for all presented results

	**Figures and Movies**	**Primary detection objective**	**Actual Magnification**	**Mode (Excitation wavelength)**	**Laser power @ sample**	**Illumination z-extent**	**z-spacing**	**xy-pixel, acquired**	**xy-pixel, reconstruct.**	**2D imaging z-slice rate**	**3D captured volume rate**
					**mW**	**µm**	**µm**	**µm**	**µm**	**Hz**	**Hz**
			(a)			*(b)*	*(c)*	*(d)*	*(e)*	*(f)*	*(g)*
Vasculature	Fig. 1c<br>(SPIM)	16 X,<br>NA = 0.8	32 X	SPIM<br>(488 nm)	0.15	3.5	1	0.203	0.203	6[#]	0.07*
	Fig. 1c<br>(SVIM)	16 X,<br>NA = 0.8	32 X	SVIM<br>(488 nm)	1	100	2	0.203	0.311	10	10
	Fig. 1d, e<br>Sup. Fig. 3-5<br>(SPIM)	20 X,<br>NA = 0.5	20 X	SPIM<br>(488 nm)	0.15	3.5	1	0.325	0.325	6[#]	0.023*
	Fig. 1d, e<br>(SVIM, 100 µm)	20 X,<br>NA = 0.5	20 X	SVIM<br>(488 nm)	1	100	2	0.325	0.498	10	10
	Fig. 1d-e<br>(SVIM, 200 µm)	20 X,<br>NA = 0.5	20 X	SVIM<br>(488 nm)	1.5	200	2	0.325	0.498	10	10
	Fig. 1d, e<br>(SVIM, 300 µm)	20 X,<br>NA = 0.5	20 X	SVIM<br>(488 nm)	2	300	2	0.325	0.498	10	10
	Fig. 1d, e<br>(SVIM, 550 µm)	20 X,<br>NA = 0.5	20 X	SVIM<br>(488 nm)	3	550	2	0.325	0.498	10	10
	Fig. 1d,<br>(Wide-field)	20 X,<br>NA = 0.5	20 X	WF-LFM<br>(488 nm)	4	Entire<br>sample	2	0.325	0.498	10	10
	Sup. Figs. 3-5	Identical parameters to Fig. 1d, e, for the respective imaging modalities									
Bacteria-squid	Fig. 2a	20 X,<br>NA = 0.5	20 X	WF-LFM<br>(488 nm)	15	Entire<br>sample	2	0.325	0.498	20	20
	Fig. 2b, c<br>Sup. Fig. 6c, d	20 X,<br>NA = 0.5	20 X	SVIM<br>(488 nm)	15	100	2	0.325	0.498	20	20
	Sup. Fig. 6b	20 X,<br>NA = 0.5	20 X	SPIM<br>(488 nm)	1	3.5	1	0.325	0.325	6[#]	0.035*
Heart-blood	Fig. 2d-g<br>(Endocardium)	16 X,<br>NA = 0.8	32 X	SVIM<br>(488 nm)	10	150	2	0.203	0.311	90	90
	Fig. 2d-g<br>(Blood)	16 X,<br>NA = 0.8	32 X	SVIM<br>(561 nm)	15	150	2	0.203	0.311	90	90
	Sup. Fig. 7a-b<br>(Endocardium)	20 X,<br>NA = 0.5	20 X	SVIM<br>(488 nm)	10	150	2	0.325	0.498	90	90
	Sup. Fig. 7d-e<br>(Blood)	20 X,<br>NA = 0.5	20 X	SVIM<br>(561 nm)	15	150	2	0.325	0.498	90	90
Brain	Fig. 2h<br>(Wide-field)	20 X,<br>NA = 0.5	20 X	WF-LFM<br>(488 nm)	0.5	Entire<br>sample	2	0.325	0.498	1	1
	Fig. 2i<br>(1p-SVIM)	20 X,<br>NA = 0.5	20 X	1p-SVIM<br>(488 nm)	0.4	100	2	0.325	0.498	1	1
	Fig. 2j<br>(2p-SVIM)	20 X,<br>NA = 0.5	20 X	2p-SVIM<br>(920 nm)	100	100	2	0.325	0.498	1	1
	Sup. Figs. 8 and 9	Identical parameters to Fig. 2h, i, j, for the respective imaging modalities									
	Sup. Fig. 2<br>(Beads)	16 X,<br>NA = 0.8	32 X	SVIM<br>(488 nm)	0.1	100	2	0.203	0.311	2	2
	Movies 1, 2, 3	Rendered from the same data as presented in Fig. 2a-c									
	Movies 4, 5	Rendered from the same data as presented in Fig. 2d-g									

Notes for Supplementary Table 2

(a) Actual magnification was determined by the ratio of the focal length of the primary detection objective over the focal length of the tube lens used.

(b) Illumination z-extent in SPIM mode was determined by the nominal thickness of the light sheet at the center of the field of view. For SVIM modes, the illumination z-extent was controlled by setting the scanning amplitude of the illumination galvo (see Methods).

(c) Spacing in z-direction for SPIM was determined by the z-step size of the acquired z-stack. Spacing in SVIM was computationally set at the onset of the light-field reconstruction.

(d) Pixel size, acquired, was determined by the physical size of the camera pixel (6.5 μm) divided by the actual magnification used during imaging.

(e) Pixel size, reconstructed, for the SVIM/LFM cases, was computationally set at the onset of the light-field reconstruction. For SPIM, the same acquired pixel size was used.

(f) The 2D imaging z-slice rate was achieved by "back-to-back" exposures, incorporating both the exposure time and camera readout time (approximately 10 ms for full camera frame). For the SPIM cases ($^{\#}$), the 2D imaging rate stated here also included the additional overhead time that was required to move the z-stage from one position to the next, which typically was the rate limiting factor. For our setup, this overhead time was about 125 ms, and when combined with the 10-ms camera readout time, set a hard limit of approximately 7.4 Hz for the z-slice rate.

(g) The 3D captured volume rate for the SPIM cases (*) were for z-stacks of depth = 100, 300, and 200 μm, for Fig. 1c, Fig. 1d, and Sup. Fig. 6b, respectively. For the SVIM/LFM cases, upon 3D reconstruction of the raw 2D data, the 2D imaging rate simply became the captured volume rate. Note that the volume rates listed here have not been normalized by the number of diffraction-limited voxels contained in each volume - this issue is addressed in Supplementary Note 2 and Supplementary Table 3.

Supplementary Table 3

Volumetric imaging rate normalized by the number of diffraction-limited voxels

		**A**	**B**	**C**	**D**
		Vasculature			Heart-blood
	(Units)	SPIM (Fig. 1d)	300-µm SVIM (Fig. 1d)	Wide-field LFM (Fig. 1d)	SVIM (Fig. 2d)
Captured image plane	µm x µm	300 x 600	300 x 600	300 x 600	200 x 400
Captured image depth	µm	300	300	400	150
Lateral extent of DL voxel	µm	0.325	2	2	1.5
Axial extent of DL voxel	µm	2.2	6	6	3
Number of captured DL pixels/plane		1.7×10^6	4.5×10^4	4.5×10^4	3.6×10^4
Number of captured DL z-planes		136	50	67	50
Number of captured DL voxels		2.3×10^8	2.2×10^6	3.0×10^6	1.8×10^6
Volume rate (*)	Volumes/s	0.023	10	10	90
Volume rate (normalized)	DL voxels/s	5.3×10^6	2.2×10^7	3.0×10^7	1.6×10^8

See Supplementary Note 2 for details. (*) Volume rate values are duplicated from Supplementary Table 2.

DL: diffraction-limited.

Supplementary Notes

Supplementary Note 1

Discussion on implementations of selective volume illumination

Selective volume illumination was implemented in our work by employing galvo-based beam scanning, to paint out the desired volume with Gaussian-focused laser light multiple times over the exposure time of each captured image (Methods, Supplementary Fig. 1). For 1p excitation, a cylindrical beam-shaping module was used to create a static 2D "sheet" of light at the sample, and the light sheet needs only to be scanned in one direction to cover the volume of interest. For 2p excitation, to achieve optimal signal rate, we used a spherically-focused beam to excite the sample[9], and scanned the beam in 2D (y & z directions, in the lab reference frame depicted in Supplementary Fig. 1), to achieve 3D volume coverage. In both implementations, the illumination focusing-NA was adjusted to be relatively low (~ 0.03 - 0.04) to achieve axially-extended focus (depth of focus ~ 500 - 250 μm), so that volume coverage along the axial direction (x, in the lab reference frame) was "automatic", i.e., without needing to scan the beam along that direction.

The main advantage of the galvo-based volume-scanning approach is the spatial precision with which we can achieve in defining the volume of interest. Just as the spatial precision of a painting is approximately given by the size of the brush, the scanned selectively-illuminated volume can be controlled to within the size of the focused illumination beam (~ 5 μm in our case), thus providing the necessary precision for our work in quantifying the difference in the images obtained with different selectively-illuminated volume extents.

An alternative to the volume-scanning approach is to illuminate the sample with a beam having a larger cross-sectional area, as large as to completely fill the volume of interest without the need to scan the illumination beam (while still minimizing illumination of the extraneous sample volume, to maintain the benefits of SVIM). This approach, implemented in a recent work[6], does give up the spatial precision with which one can achieve the selectively-illuminated volume, as a larger "brush" is used to fill in the volume of interest. A large beam, with a cross-sectional diameter of ~ 100-μm or more, in either a focused or collimated state, would necessarily have their boundaries less sharp than a focused beam with diameter of 5-μm as used in our case. Practically, such beams of diameter ~ 100-μm or more would have their blurred boundaries extending over several tens of microns or more. While we deem this precision is not enough for our work presented here, we envision that for many biological imaging applications, the precision of ~ several tens of microns is adequate, and thus the volume-filling strategy to implement SVIM could be used in such scenarios.

The volume-scanning approach to implement SVIM has several disadvantages. First is its relative complexity and cost, due to the need for the galvo scanners and associated electronics and software to

control the scanning. Second, the scanning could in principle restrict the volumetric imaging rate of the setup: the highest imaging rate would be limited to the fastest time needed to paint out the volume once. We consider this last issue with some back-of-envelope calculations below.

For the relatively small scanning angles of < 2-3 degrees employed in our SVIM setup, the galvo could reach a maximal line-scanning rate of 1.5 kHz, or line-scanning period of 0.67 ms. Thus, for the 1D scanning employed with 1p excitation, the entire volume, with a depth up to 1 mm, could be painted out within 0.67 ms, yielding ~ 1500 volumes/s as the fastest possible volumetric imaging rate. This rate is over one order of magnitude higher than the full-frame imaging rate of current state-of-the-art sCMOS cameras, which typically top out at 100 frames/s. For the 2D scanning employed with 2p excitation, a volume of depth ~ 300 μm could be painted out with ~ 100 lines (with a nominal cross-sectional diameter of 6 μm), thus achieving a single-volume scanning time of 67 ms, which is equivalent to ~ 15 volumes/s as the fastest volumetric imaging rate. If the galvo in our setup is replaced with a resonant scanner, which can scan ~ 10 times faster, the maximum volumetric rate could be as high as 150 volumes/s. Thus, we see that quite high imaging rates are still possible with the volume-scanning approach: 100 volumes/s for 1p (limited by camera speed), and 15-100 volumes/s for 2p excitation. Furthermore, higher imaging rates with lower exposure times require higher optical excitation intensities to maintain useful signal-to-noise ratios. However, concerns about photo-damage to the biological samples would often place a hard restriction on the light intensity that can be used. This is a particular concern with 2p excitation, given the substantially lower excitation cross-section for 2p absorption. Thus, in practice, the useful volumetric imaging rate of SVIM is restricted more by the threshold of photo-damage than by the constraints of the volume-scanning strategy.

Supplementary Note 2

Volumetric imaging rate normalized by the number of diffraction-limited voxels

In the comparison of the volumetric imaging rate of SVIM, and LFM in general, with SPIM, it is instructive to also consider the imaging rate normalized by the number of diffraction-limited voxels, due to the large difference in resolution of SVIM and SPIM. This exercise will provide insights into the trade-offs involved between high-resolution and high-speed imaging.

The diffraction-limited (DL) voxel is defined as the smallest useful rectangular volume unit that is captured, extending in each of the spatial directions by an amount, following Nyquist criterion, equal to half of the spatial resolution achieved along that direction. The spatial resolution is determined by either the optical resolution of the combined imaging and reconstruction pipeline, or the experimental spatial sampling during imaging (i.e., the lateral pixel size and z-slice thickness), whichever that is larger.

For concreteness, we calculated the normalized volumetric imaging rate for several representative imaging scenarios and present the results in Supplementary Table 3 (refer to Supplementary Table 2 for the corresponding imaging and reconstruction parameters). For SPIM imaging of the vasculature (Fig. 1d), the expected optical resolution for detection NA = 0.5 is ~ 0.51 μm laterally and ~ 4.4 μm axially while the spatial sampling is lateral pixel = 0.325 μm and z-slice thickness = 1 μm. Thus we arrived at the lateral extent = 0.325 μm and axial extent = 2.2 μm for the DL voxel. (For the SPIM optical resolution calculation, we used the formulas: lateral resolution = $\lambda/(2*NA)$, axial resolution = $2*\lambda/(NA^2)$, with $\lambda = 0.55$ μm.) For the SVIM/LFM imaging cases, following[4] and the experimental optical parameters, we estimated the resolution to be 4 μm laterally and 12 μm axially for the SVIM/LFM vasculature reconstruction (Fig. 1d), and 3 μm laterally and 6 μm axially for the SVIM heart-blood reconstruction (Fig. 2d). The latter case was experimentally confirmed (Supplementary Fig. 2). For all SVIM/LFM cases, the DL voxel sizes were determined by the optical resolution, as they were larger than the spatial sampling (see Supplementary Table 2).

From the sizes of the DL voxels, we straightforwardly calculated the DL-voxel-normalized volumetric imaging rate for the different imaging scenarios, shown in Supplementary Table 3. In our work, we did not push the experimental conditions to achieve the fastest possible imaging rate, for either SPIM or SVIM/LFM. However, the imaging rates shown in the table below could still be usefully compared to each other as "typical" performances, and more importantly to demonstrate the parameters and relationships that are involved in determining the normalized volume rate. The vasculature results shown for SPIM, 300-μm SVIM, and wide-field LFM cases (labeled as A, B, C in the table) were from imaging the same sample, so they can be directly compared with each other. The heart-blood SVIM result (labeled as D in the table) represents a scenario where SVIM enabled a high volumetric imaging rate, normalized by the number of DL voxels or not, that was not reachable with conventional SPIM, as discussed in the main text.

From the results shown in Supplementary Table 3, we can make a few observations concerning the trade-offs involved in choosing between high-resolution or high-speed imaging. First, the 3-orders of magnitude higher volume rate of SVIM/LFM compared to SPIM gets reduced to a factor of ~ 4 after the rates are normalized by the number of DL voxels captured (comparing A and B in the table). This is not surprising, since the higher resolution achieved with SPIM means that many more DL voxels are captured with SPIM than with SVIM/LFM. Thus, we see that SVIM/LFM achieves high imaging rate by trading away resolution, capturing a reduced number of DL voxels while still covering the same sample volume. However, the speed advantage of SVIM/LFM does not come entirely from capturing a reduced number of DL voxels. It also comes from the fact that the entire volume is imaged in a single snapshot, eliminating the overhead time needed in SPIM to go from one imaged z-plane to the next (see note (f) of Supplementary Table 2). Thus, the larger the z-extent of the volume of interest, the better performance SVIM/LFM will have in terms of the normalized volumetric imaging rate (comparing A, B, and C in the table). This speed benefit of thicker-z-extents of SVIM/LFM of course comes with a cost, namely larger z-extents will have lower image contrast and effective resolution, depending on the sample properties, as we have shown in our work here.

References for Supplementary Information

1. Levoy, M., Ng, R., Adams, A., Footer, M. & Horowitz, M. Light field microscopy. *ACM Transactions on Graphics (TOG)* **25**, 924–934 (2006).
2. Broxton, M. *et al.* Wave optics theory and 3-D deconvolution for the light field microscope. *Optics Express* **21**, 25418 (2013).
3. Edelstein, A. D. *et al.* Advanced methods of microscope control using μManager software. *J Biol Methods* **1**, (2014).
4. Cong, L. *et al.* Rapid whole brain imaging of neural activity in freely behaving larval zebrafish (Danio rerio). *eLife Sciences* **6**, e28158 (2017).
5. Skocek, O. *et al.* High-speed volumetric imaging of neuronal activity in freely moving rodents. *Nat Methods* **15**, 429–432 (2018).
6. Wagner, N. *et al.* Instantaneous isotropic volumetric imaging of fast biological processes. *Nat Methods* **16**, 497–500 (2019).
