# Supplementary material for: High-contrast, synchronous volumetric imaging with selective volume illumination microscopy
Source: Commun Biol. 2020 Feb 14;3:74. doi: 10.1038/s42003-020-0787-6 (PMC7021898; doi:10.1038/s42003-020-0787-6)
Supplement: Supplementary file 11 — Description of Additional Supplementary Files [file 42003_2020_787_MOESM11_ESM.pdf]

## Description of additional supplementary items

**Supplementary data_Fig 2b.xlsx:** data underlying the plot shown in Fig. 2b.

**Supplementary data_Fig 4def.xlsx:** data underlying the plots shown in Fig. 4d,e,f.

**Supplementary Movie 1**

**Raw light-field-image movie of the bacterial flow around the squid light organ, recorded with SVIM**

Movie depicts the raw, unprocessed 2D-light-field images of the flow of fluorescently-labeled *Vibrio fischeri* bacteria around the light organ of the Hawaiian bobtail squid *Euprymna scolopes*, during early stage of colonization. Time series were acquired at 20 frames $s^{-1}$, and the movie playback frame rate was set at the same rate. Note the high contrast and resolution achieved in these raw light-field SVIM images, which enabled following the 3D motion of individual bacterium. A single frame of the movie was shown in Fig. 3b. Scale bar, 100 μm.

**Supplementary Movie 2**

**Comparison of reconstructed movie of the bacterial flow around the squid light organ, recorded with wide-field LFM versus with SVIM**

Movie depicts the side-by-side comparison of the reconstructed movies recorded with wide-field LFM versus with SVIM, of the flow of fluorescently-labeled *Vibrio fischeri* bacteria around the light organ of the Hawaiian bobtail squid *Euprymna scolopes*, during early stage of colonization. Each 4D dataset (time-series, z-stack) was reconstructed with the same parameters from the raw data (Methods), then normalized in intensity levels with 0.4% pixel saturation for display purposes here, and finally a z-projection was taken based on average values. Time-series were acquired at 20 frames $s^{-1}$, and movie playback frame rate was set at the same rate. Raw time-series for the two modalities were recorded sequentially in time, and frame-matched here to facilitate comparison. Note the high contrast and single-bacterium resolution achieved with SVIM, compared with the low contrast of the wide-field LFM reconstruction. Scale bar, 100 μm.

**Supplementary Movie 3**

**3D flow fields of bacteria around the squid light organ, recorded with SVIM**

Movie depicts the flow fields of fluorescently-labeled *Vibrio fischeri* bacteria around the light organ of the Hawaiian bobtail squid *Euprymna scolopes*, tracked from the 3D-reconstructed light-field rendering. Light-field images were acquired at 20 frames $s^{-1}$, yielding 3D volumetric rate of 20 volumes $s^{-1}$ after reconstruction, of volume ~ 600 x 600 x 100 (depth) $\mu m^3$. Movie playback frame rate was set to 20

frames $s^{-1}$. Gray-scale image in the background is the average intensity projection of the 3D reconstruction. Individual fluorescent bacterium was computationally tracked, and tracks are shown color-coded by z position. Analysis of the tracks provided a quantitative description of the 3D bacterial flow fields, as shown in Fig. 2c and Supplementary Fig. 6c, d. Scale bar, 100 μm.

**Supplementary Movie 4**

**3D blood flow and endocardium motion of the entire larval zebrafish beating heart**

Movie of fluorescently-labeled endocardium (white) and blood cells (red) in a beating heart of a live 5-dpf zebrafish larva, 3D-rendered following the light-field reconstruction. Acquisition was at 90 volumes $s^{-1}$, and movie playback frame rate was slowed by 5.5 times. Representative blood cells' trajectories through the heart were manually tracked and quantified (color of the trajectories depicts speed). During the movie, the endocardium channel was turned off at several time points to aid visualization of the blood cells. The high synchronous volumetric imaging rate and volume coverage enabled imaging and tracking of the 3D blood flow, at single-blood-cell resolution throughout the cardiac beating cycle, which was about 450 ms. Still frames from the movie were depicted in Fig. 3d, e, g. Scale bar, 50 μm.

**Supplementary Movie 5**

**3D trajectories of individual blood cells flowing through the larval zebrafish beating heart**

Movie depicts the representative trajectories of blood cells flowing through the larval zebrafish beating heart, as seen while rotating the imaged cardiac volume about the y-axis. Note the substantial extent of the motion along the z direction, and the non-uniform 3D trajectories, of the blood cells. Acquisition was at 90 volumes $s^{-1}$, and movie playback frame rate was slowed by 5.5 times. Centroids of blood cells are shown as black-colored spheres. Each individual blood cell trajectory is represented in a single color to aid visualization. A 3D-cropped still frame of the movie was shown in Fig. 3f. Scale bar, 50 μm.

**Supplementary Movie 6**

**Comparison of SVIM and wide-field LFM in imaging the endocardium of the zebrafish beating heart**

Side-by-side comparison of SVIM (right panel) and wide-field LFM (left panel) in imaging the fluorescently-labeled endocardium of the beating heart of a 5-dpf zebrafish larvae. Acquisition was at 90 volumes $s^{-1}$, and movie playback frame rate was slowed by 5.5 times. The 3D reconstructions are shown as an averaged-intensity projection in z. Still frames from the movie are presented in Supplementary Fig. 7a, b, and the quantitative contrast analysis presented in Fig. 7c. To facilitate comparison across the heart

beating cycle, the movies taken with SVIM and wide-field LFM were frame-synchronized to represent a similar beating sequence, taking advantage of the periodic nature of the beating heart (see Supplementary Fig. 7 for more detail). Scale bar, 50 μm.

**Supplementary Movie 7**

**Comparison of SVIM and wide-field LFM in imaging the blood cells of the zebrafish beating heart**

Side-by-side comparison of SVIM (right panel) and wide-field LFM (left panel) in imaging the fluorescently-labeled blood cells flowing through the beating heart of a 5-dpf zebrafish larvae. Acquisition was at 90 volumes $s^{-1}$, and movie playback frame rate was slowed by 5.5 times. The 3D reconstructions are shown as an averaged-intensity projection in z. Still frames from the movie are presented in Supplementary Fig. 7d, e, and the quantitative contrast analysis presented in Fig. 7f. To facilitate comparison across the heart beating cycle, the movies taken with SVIM and wide-field LFM were frame-synchronized to represent a similar beating sequence, taking advantage of the periodic nature of the beating heart (see Supplementary Fig. 7 for more detail). Scale bar, 50 μm.
